# Mouse mesoderm-specific transcript inhibits adipogenic differentiation and induces trans-differentiation into hepatocyte-like cells in 3T3-L1 preadiocytes

**DOI:** 10.1186/s13104-022-06051-x

**Published:** 2022-05-10

**Authors:** Yoshito Kadota, Takashige Kawakami, Masao Sato, Shinya Suzuki

**Affiliations:** Faculty of Pharmaceutical Sciences, Tokushima Bunri University, 180 Yamashiro-cho, Tokushima, Japan

**Keywords:** Mesoderm specific transcript, Adipocyte, Lipid accumulation, Trans-differentiation, Hepatocyte

## Abstract

**Objective:**

The mesoderm-specific transcript (*Mest*) is an imprinted gene that is transcribed from the paternal allele. It is a marker of adipose tissue expansion; however, it is uncertain whether *Mest* expression promotes or suppresses adipogenic differentiation. To elucidate the effects of *Mest* expression on adipogenic differentiation, we transfected an expression vector or siRNA for mouse *Mest* into 3T3-L1 mouse preadipocyte cell line.

**Results:**

In differentiated 3T3-L1 adipocytes, *Mest* overexpression decreased lipid accumulation. Conversely, gene silencing of *Mest* increased the accumulation of lipid droplets in adipocytes. These results demonstrate that Mest negatively regulates adipocyte differentiation. Further, Mest induced trans-differentiation of 3T3-L1 cells into hepatocytes, and its overexpression induced the expression of hepatocyte marker genes, including albumin and α-fetoprotein. In the presence of dexamethasone, the forced expression of the *Mest* caused morphological changes in 3T3-L1 cells. Cells were flat and polygonal shapes, with an increased accumulation of intracellular glycogen and other features that are typical of hepatocytes. Therefore, Mest inhibits adipogenic differentiation of 3T3-L1 preadipocytes by inducing hepatocyte trans-differentiation.

**Supplementary Information:**

The online version contains supplementary material available at 10.1186/s13104-022-06051-x.

## Introduction

The mesoderm-specific transcript (*Mest*) is an imprinted gene that is transcribed from the paternal allele and is expressed in the embryonic and extraembryonic mesoderm [1–4]. The Mest protein is a member of the α/β-hydrolase-fold superfamily, but its activity remains unknown [2].

The *Mest* mRNA levels are markedly upregulated in white adipose tissue in obese mice, and its increased expression correlates with the size of adipocytes [5–7]. In transgenic mice, Mest overexpression increases adipocyte size [5], while *Mest* knockout suppresses adipose tissue expansion in mice fed with a high-fat diet [8]. In vitro, *Mest* is upregulated during the differentiation of 3T3-L1 preadipocytes into adipocytes [9]. *Mest* overexpression promotes the differentiation of 3T3-L1 preadipocytes into adipocytes, and *Mest* silencing suppresses this differentiation [5, 10]. *Mest* knockout enhances the intracellular lipid accumulation in mouse mesenchymal progenitor cells that were treated with inducers of adipogenic differentiation [8]. Additionally, siRNA for human *MEST* promoted the differentiation of human adipose-derived stem cells into adipocytes [11]. To better understand these diverse activities, we re-evaluated the effects of *Mest* overexpression and silencing on the adipogenic differentiation of 3T3-L1 preadipocytes.

## Main text

### Materials and methods

#### Cell culture and establishment of 3T3-L1 cell lines that stably express mest

We employed the pcDNA3.1 plasmid vector containing both the *Mest* and a neomycin resistance gene [12]. The 3T3-L1 cells (5 × 10^5^) were seeded onto 60-mm dishes one day prior to transfection in Dulbecco’s modified Eagle’s medium (DMEM) supplemented with 10% Calf serum (CS) (Sigma-Aldrich, St. Louis, MO, USA). The cells were treated with a complex of Lipofectamine 3000 (Life Technologies, Carlsbad, CA, USA) and 5 µg of plasmid DNA. After 24 h of incubation, the cells were trypsinized and seeded into 100-mm dishes; then, neomycin-resistant cells were selected with 1 mg/mL G418. The 3T3-L1 preadipocytes, mock-transfected cells, and *Mest* expression vector-transfected 3T3-L1 cells, designated 3T3-L1-Mest, were maintained in DMEM supplemented with 10% CS at 37 °C in the presence of 5% CO_2_.

#### RNA isolation and gene expression analysis using by RT-PCR

The total cellular RNA was isolated using RNAiso Plus (Takara Bio, Otsu, Japan). The RNA from each sample was reverse-transcribed using a High-Capacity cDNA RT kit (Applied Biosystems, Foster City, CA, USA). PCR was performed within a linear range of amplification using the primer sets indicated in Additional file 1: Table S1. The PCR products were resolved on 2% agarose gel electrophoresis and visualized with ethidium bromide using a LAS-4000 mini image analyzer (Fujifilm, Tokyo, Japan).

#### Immunoblotting for expressed Mest protein

The cells were digested using mammalian protein extraction reagent (Pierce Biotechnology, Rockford, IL, USA), which contained a protein inhibitor cocktail (Complete Mini; Roche Diagnostics. K.K., Basel, Switzerland) After centrifugation at 20400*g* for 15 min at 4 °C, aliquots of the supernatants were treated with 25 mM mercaptoethanol, boiled at 100 °C for 2 min, and 20 μg of protein from each sample was resolved using a sodium dodecyl sulfate–polyacrylamide gel electrophoresis, in a 10% gel. The proteins were transferred from the gel to a polyvinylidene difluoride membrane in a basic transfer buffer (48 mM Tris, 39 mM glycine, and 20% methanol, pH 9.2) using an electroblotter. Proteins were visualized by immunostaining with a primary antibody (either a goat anti-Mest antibody (1:1,000) or a rabbit anti-β-actin IgG (1:5,000) (Abcam Ltd., Cambridge, UK)), a secondary antibody (either a horseradish peroxidase (HRP)-conjugated anti-goat IgG (Millipore, Billerica, MA, USA) or HRP-conjugated anti-goat IgG antibody (Abcam Ltd.)), and a chemiluminescent substrate (Millipore, Billerica, MA, USA) using an LAS-4000 mini image analyzer. Prestained protein markers (Bio-Rad, Hercules, CA, USA) were used as the standard molecular mass proteins.

#### Differentiation induction toward adipocytes and hepatocytes

The cells were seeded at a density of 3 × 10^4^ cells/cm^2^ and precultured for two days. Adipogenic differentiation of preadipocytes was induced on day 0 by replacing the original medium with DMEM containing 10% fetal bovine serum (FBS) (Sigma-Aldrich) supplemented with an adipogenic cocktail (1 µg/ml insulin, 1 μM dexamethasone (DEX), and 0.5 mM 3-isobutyl-1-methylxanthine (IBMX) (Sigma-Aldrich). After two days (day 2), the culture medium was changed to DMEM containing 10% FBS supplemented with 1 µg/ml insulin, and the cells were cultured for two more days. On day 4, the medium was replaced with DMEM containing 10% FBS, subsequently the cells were cultured until day 8. For trans-differentiation into hepatocytes, the cells were treated with 1 µM DEX in DMEM containing 10% FBS for four days, and then the medium was replaced and cells were treated DMEM containing 10% FBS for four more days.

#### Oil Red O staining

Lipid accumulation was evaluated by measuring Oil Red O retention. The cells were fixed with 4% paraformaldehyde (FUJIFILM Wako Pure Chemical, Osaka, Japan) and stained with 3 mg/mL Oil Red O (Sigma-Aldrich) in 60% 2-propanol. To quantify intracellular lipid accumulation, the absorbance of Oil Red O was measured at 520 nm using a microplate spectrophotometer.

#### Knockdown of Mest by siRNA

We transfected the cells with stealth siRNAs targeting mouse *Mest* mRNA and a RNAi negative control (Life Technologies, Carlsbad, CA, USA) at a final concentration of 20 nM using Lipofectamine RNAi MAX (Life Technologies Carlsbad, CA, USA). The sequences of the siRNAs and negative control RNAs are shown in Additional file 2: Table S2.

#### Periodic acid-Schiff (PAS) stain for glycogen

The cells were fixed in 4% paraformaldehyde. After rinsing in distilled water, the samples were oxidized in 0.5% periodic acid (FUJIFILM Wako Pure Chemical) for 10 min and then reacted with Schiff’s reagent (Merck KGaA, Darmstadt, Germany) for 30 min. Cells were rinsed again, this time in tap water for 5 min. Samples were counterstained with Mayer’s hematoxylin for 1 min and rinsed with distilled water.

#### Measurement surface area of cells

The cells were trypsinized and resuspended in DMEM + 10% CS. The cell suspension was then applied to a cell counter plate. Images were obtained using the TrueChrome II (TUCSEN Photonics, Fuzhou, China). The surface area of the cells in the images was calculated using the software functions of the adjusted threshold and analyzed using ImageJ (ImageJ: http://imagej.nih.gov/ij).

### Statistical analysis

BellCurve for Excel (Social Survey Research Information, Tokyo, Japan) was used for the statistical analysis. Data sets were compared for significant differences by one-way analysis of variance using the Dunnett’s test or the paired Student’s *t*-test.

### Results

#### Expression of recombinant mouse Mest and Mest protein in transformed 3T3-L1 cells

**Fig. 1:**
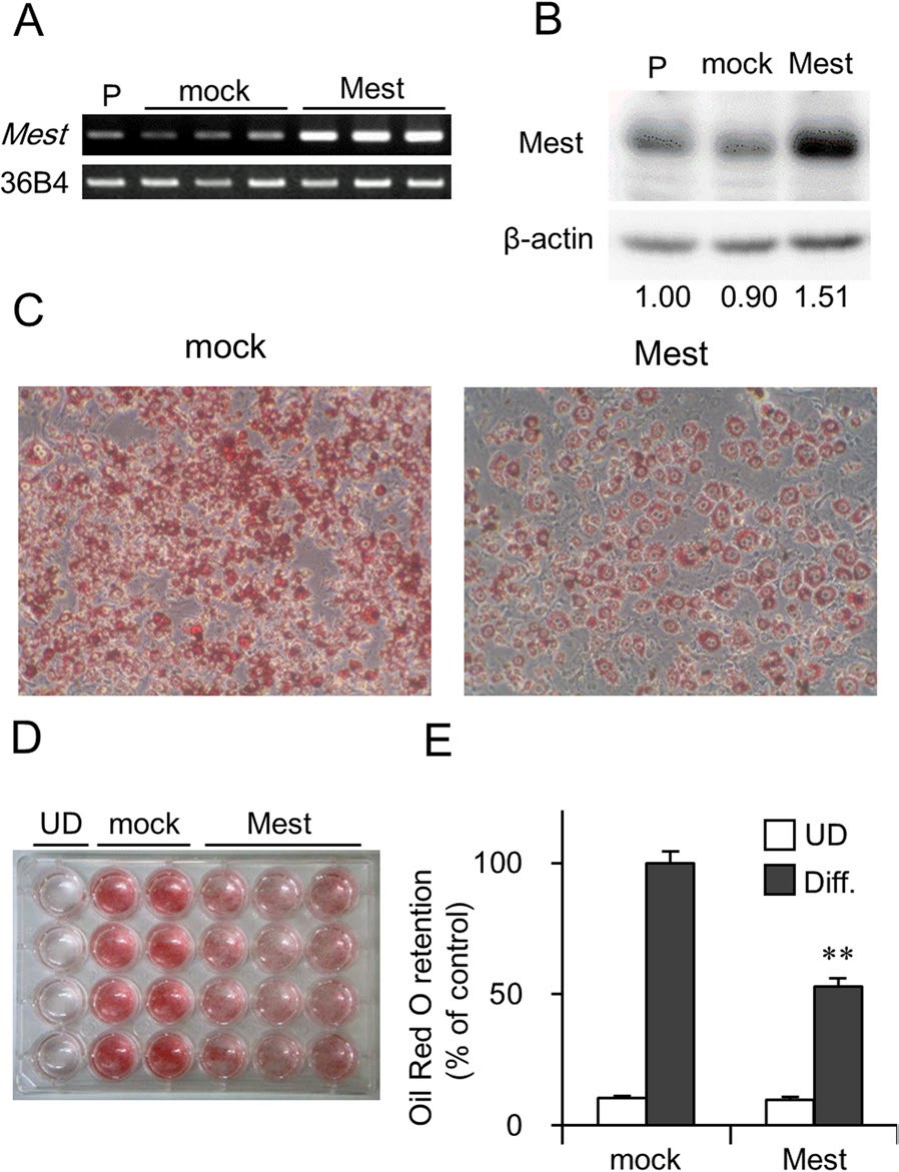
*Mest* overexpression reduces lipid accumulation in 3T3-L1 cells. **A** The expression levels of *Mest* and 36B4 mRNA as housekeeping gene in 3T3-L1 parent cells (P), 3T3-L1 mock-transfected cells (mock), and 3T3-L1-Mest (Mest). **B** Western blot analysis of the expression of Mest and β-actin as internal control in 3T3-L1 parent cells (P), 3T3-L1 mock-transfected cells (mock), and 3T3-L1-Mest (Mest). The values under each lane indicated relative density of the band of Mest protein normalized to β-actin. **C** Representative images of lipid accumulation by Oil Red O staining in 3T3-L1 mock-transfected cells, and 3T3-L1-Mest (Mest). The differentiation was induced by treatment of a standard adipogenic cocktail and additional reagent, 10 μM pioglitazone, which promotes the differentiation and makes it easy to evaluate of lipid accumulation. **D** Representative photographs of 24-well cell culture plate of Oil Red O staining in undifferentiated 3T3-L1 cells (UD), mock-transfected cells (mock), and *Mest*-overexpressing cells (Mest). **E** Quantification of Oil Red O retention in undifferentiated (UD) and differentiated (Diff.) 3T3-L1 mock and 3T3-L1 Mest. Values are presented as the mean ± SD (*n* = 8). **P < 0.01 vs 3T3-L1 mocked cells

We compared *Mest* expression levels between 3T3-L1 cells, 3T3-L1-Mest, and mock-transfected cells. While the expression level of *Mest* in mock-transfected cells was similar to that of the parent cells, *Mest* mRNA was significantly expressed in the 3T3-L1-Mest cells (Fig. 1A and Additional file 3: Fig. S1A, B). Immunoblotting analysis with an anti-Mest protein antibody revealed a significant increase in Mest protein, an approximately 53-kDa protein in 3T3-L1-Mest cells, compared with the mock-transfected cells (Fig. 1B and Additional file 3: Fig. S1C, D).

#### Overexpression of *Mest* decreases in lipid accumulation in 3T3-L1 adipocytes

To evaluate the effect of *Mest* gene overexpression on adipogenic differentiation of 3T3-L1 cells, the retention of Oil Red O in lipid droplets was measured. The quantity of Oil Red O in differentiated 3T3-L1-Mest adipocytes was half of that in mock-transfected cells (Fig. 1C–E).

#### Silencing of Mest gene stimulates adipocyte differentiation

**Fig. 2:**
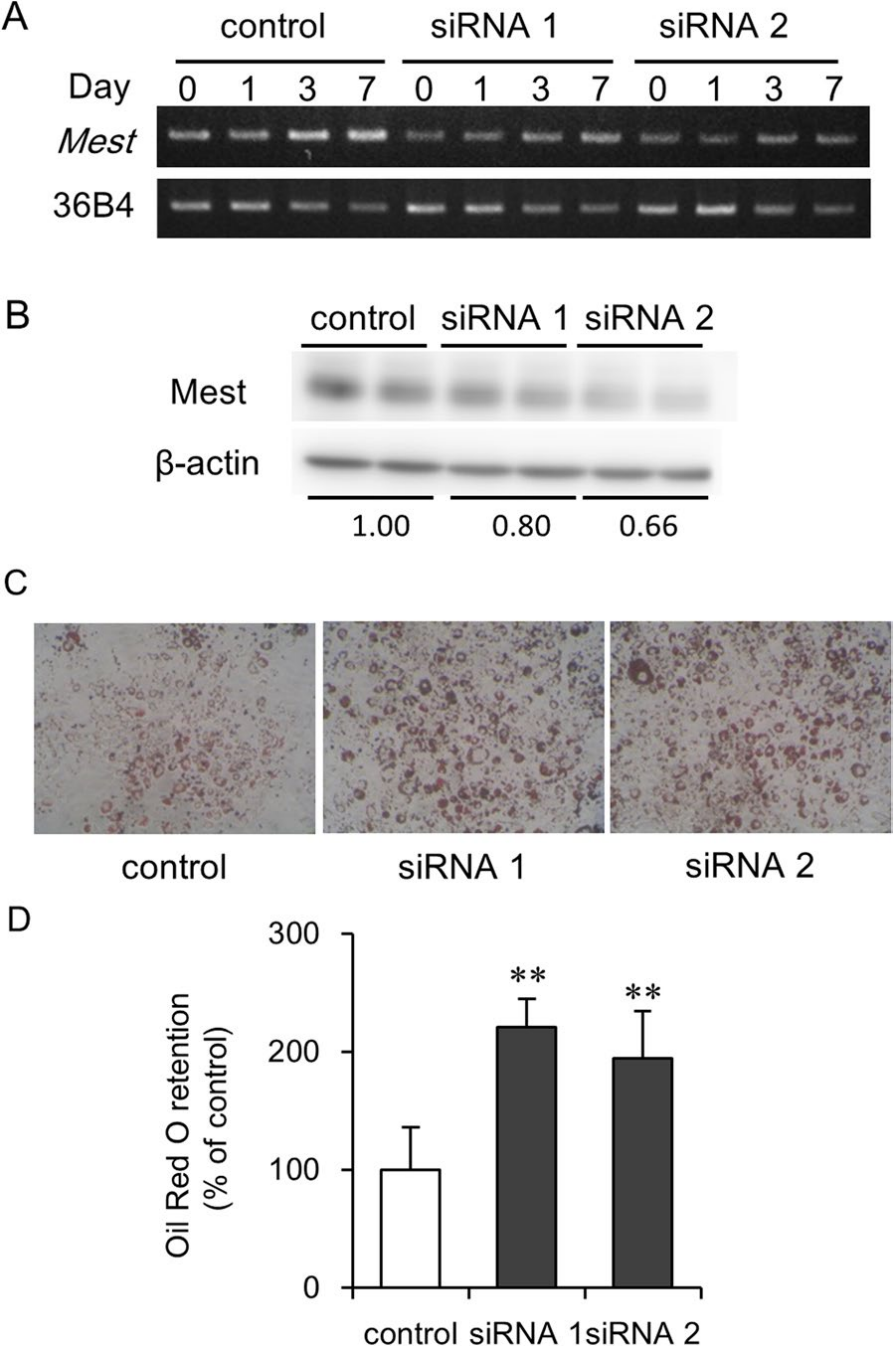
Knockdown of *Mest* by siRNA enhances adipogenic differentiation of 3T3-L1 cells. **A** The expression levels of *Mest* and 36B4 mRNA as housekeeping gene in 3T3-L1 cells, which were transfected with siRNAs for *Mest* (siRNA 1 and 2) or with RNAi negative control. **B** Western blot analysis of the expression of Mest and β-actin as internal control in 3T3-L1 cells, which were transfected with siRNAs for *Mest* (siRNA 1 and 2) or with RNAi negative control. The values under bands indicated relative density of the bands of Mest protein normalized to β-actin. **C** Representative images and (**D**) quantification of lipid accumulation by Oil Red O staining in 3T3-L1 cells, treated with control RNA and siRNAs for *Mest* (siRNA 1 and 2). Values are presented as the mean ± SD (*n* = 5). **P < 0.01 vs control RNA treated-3T3-L1 cells

To analyze the loss of function of the Mest on adipocyte differentiation, we used two siRNAs targeting *Mest* mRNA. The *Mest* mRNA expression levels increased on day 3 and remained steady until day 7 during adipocyte differentiation in 3T3-L1 cells (Fig. 2A), as reported previously [9]. Both siRNAs 1 and 2 repressed the upregulation of Mest mRNA and protein expression (Fig. 2A, B, and Additional file 4: Fig. S2A–D). Treatment with the siRNAs for the *Mest* significantly increased lipid droplets in 3T3-L1 adipocytes (Fig. 2C, D).

#### Mest induces cell enlargement of 3T3-L1 preadiocytes

**Fig. 3:**
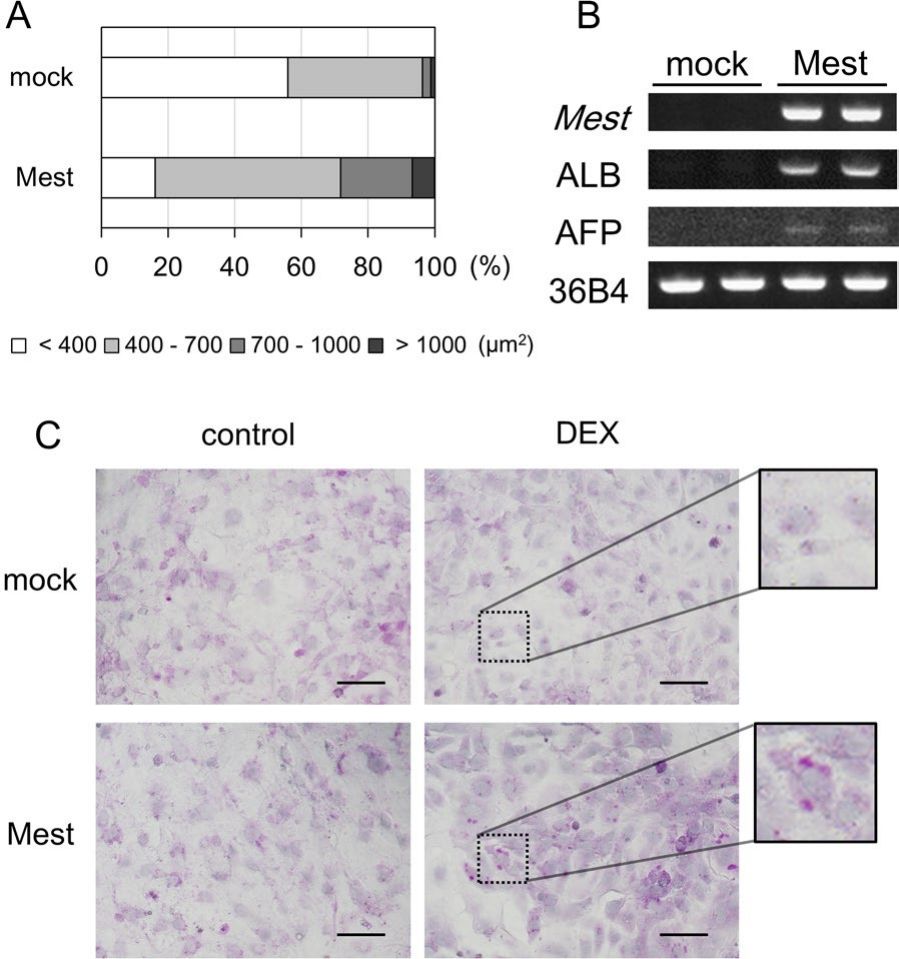
*Mest* induces a cell enlargement and trans-differentiation of 3T3-L1 cells into hepatocyte-like cells. **A** The distribution ratio of 3T3-L1 mock-transfected cells and *Mest* overexpression cells with different cell surface area (µm^2^). The individual cell surface area was obtained from eight representative fields of view. **B** The expression levels of *Mest*, albumin (ALB) and a-fetoprotein (AFP), which are hepatocyte specific marker genes, and 36B4 as housekeeping gene in 3T3-L1 mock-transfected cells (mock), and 3T3-L1-Mest (Mest). **C** 3T3-L1 mock-transfected cells (mock) and 3T3-L1-Mest cells (Mest) were treated with 1 µM dexamethasone (DEX) in DMEM containing 10% FBS for four days, and then the medium was replaced and treated with DMEM containing 10% FBS for four more days. Intracellular glycogen accumulation and nuclear were stained by Periodic Acid Schiff (PAS, purple-red) and hematoxylin (purple-blue), respectively. The squares indicate high-magnification image of the dotted area. (Scale bar = 100 µm)

While more than half of 3T3-L1-mock cells hadcell surface area less than 400 µm^2^, most of 3T3-Mest preadipocytes showed over 400 µm^2^ (Fig. 3A). These results suggested that the Mest causes cell enlargement in 3T3-L1 preadipocytes, but not adipocytes.

#### Mest induces trans-differentiation of 3T3-L1 preadipocytes toward hepatocyte-like cells

Although mesenchymal stem cells (MSCs) are multipotent cells, that can differentiate into a variety of cell types, including osteoblasts, chondrocytes, and adipocytes [13, 14]. The MSCs can also be transdifferentiated into hepatocytes [15]. Wnt/β-catenin signaling was suppressed during the trans-differentiation of human MSCs toward hepatocytes [16, 17]. This suppression of Wnt/β-catenin signaling enhanced the hepatic differentiation of MSCs [16–18]. The 3T3-L1 preadipocytes are derived from mouse embryonic fibroblasts [19] and are differentiated into adipocyte-like cells by treatment with an adipogenic cocktail [20]. 3T3-L1 cells are that are similar to the MSC lineage and can differentiate into osteoblasts [21–24]. Jung et al*.* reported that Mest inhibits Wnt signaling through the regulation of LRP6 glycosylation [10]. We hypothesized that *Mest* overexpression in 3T3-L1 cells induced the dedifferentiation of 3T3-L1 preadipocytes into MSCs or the trans-differentiation into hepatocytes. We confirmed the mRNA expression levels of the hepatocyte marker genes, including albumin (ALB) and α-fetoprotein (AFP), in 3T3-L1 mock and Mest cells. Increased expression of ALB mRNA and weak expression of AFP mRNA were observed in 3T3-L1-Mest cells (Fig. 3B, and Additional file 5: Fig. S3A–D). DEX was shown to induce trans-differentiation of MSCs into hepatocytes [25]. In addition, DEX induced polygonal and cuboidal morphology in rat primary hepatocytes [26]. 3T3-L1-Mest and mock-transfected cells were treated with 1 μM DEX. In the presence of DEX, forced expression of the *Mest* gene caused morphological changes in 3T3-L1 cells with flat and polygonal shapes and intracellular glycogen accumulation, which are both features of liver cells (Fig. 3C).

## Discussion

Takahashi et al*.* demonstrated that the *Mest* is a marker of adipose cell size in mice [5]. We also confirmed that the expression levels of the *Mest* were positively correlated with adipocyte size in mouse adipose tissue [27, 28]. Previous studies demonstrate that *Mest* overexpression in 3T3-L1 preadipocytes promotes adipocyte differentiation [5]; however, when the overexpression of *Mest* gene was induced, differentiation was suppressed rather than promoted (Fig. 1C–E). These contradictory results may be due to differences in the copy number of the exogenous *Mest,* compared between the retrovirus vector and our plasmid vector.

Jung et al*.* showed that shRNA for the mouse *Mest* suppressed differentiation of 3T3-L1 cells [10]. In contrast, Anunciado-Koza et al*.* reported that *Mest* knockout promoted the differentiation of mouse stem cells [8]. Knockdown of human *MEST* facilitated the differentiation of human multipotent adipose-derived stem cells [11]. Even though Karbiener et al. suggest the possible differences in species-specific regulation between humans and mice, our data indicates mouse *Mest* (Fig. 2B, C) support the results of gene silencing of human *MEST* in human stem cells. Hasegawa et al*.* reported that the overexpression of human *MEST* enhanced the expression of stem cell markers and the multi-differentiation capacity, and knockdown of *MEST* inhibited the expression of stem cell markers and promoted differentiation of periodontal ligament stem cells [29]. In mouse adipose tissue, the expression level of *Mest* is decreased during the lactation period, when adipocyte differentiation and maturation should become more prolific [7]. We showed that overexpression of *Mest* induced cell enlargement in 3T3-L1 preadipocytes (Fig. 3A). The function of Mest in adipocyte differentiation and adipocyte expansion should be considered separately.

Activated Wnt signaling downregulates the differentiation of MSCs into adipocytes and hepatocytes [30]. Thus, it seems consistent that the forced expression of *Mest* upregulates adipogenesis through the inhibition of Wnt signaling. However, the suppression of Wnt/β-catenin signaling also enhances hepatic differentiation of MSCs [16–18]. The different levels and/or the timing of *Mest* expression may be involved in the differentiation of 3T3-L1 cells into either adipocytes or non-adipocyte lineage, hepatocyte-like cells. The differentiation of 3T3-L1 cells into hepatocytes induced by Mest could prevent the preadipocytes from differentiating into adipocytes. Additionally, yet to be elucidated functions of Mest, other than Wnt inhibition, may exist.

## Limitations

This study evaluated the effects of *Mest* in 3T3-L1 cells on adipogenic differentiation using Oil Red O staining and on hepatocyte trans-differentiation using RT-PCR and morphology observation. To measure the expression levels of differentiation markers for gene expression, different of techniques should be employed, including quantitative RT-PCR.

## Supplementary Information

**Additional file 1:** **Table S1**. Primer information.

**Additional file 2:** **Table S2**. Sequence information of siRNAs targeting mouse Mest.

**Additional file 3: Figure S1.** (A) and (B) Original images of agarose gel electrophoresis from Figure 1 A.(C) and (D) Original images of western immunoblot data from Figure l B. M, marker; P, parent cells.

**Additional file 4: Figure S2**. (A) and (B) Original images of agarose gel electrophoresis from Figure 2A. (C) and (D) Original images of western immunoblot data from Figure 2B. M, marker; P, parent cells. M, marker; NS, nonspecific bands.

**Additional file 5: Figure S3**. (A), (B), (C) and (D) Original images of agarose gel electrophoresis from Figure 3B. M, marker.

## Data Availability

The datasets useed and/or analyzed during the current study are available from the corresponding author on reasonable request.

## Abbreviations

Mest: Mesoderm-specific transcript
DMEM: Dulbecco’s modified Eagle’s medium
CS: Calf serum
FBS: Fetal bovine serum
IBMX: 3-Isobutyl-1-methylxanthine
RT-PCR: Reverse transcription-polymerase chain reaction
HRP: Horseradish peroxidase
DEX: Dexamethasone
ALB: Albumin
AFP: α-Fetoprotein
PAS: Periodic acid-Schiff
MSCs: Mesenchymal stem cells
