# Supplementary material for: Mouse mesoderm-specific transcript inhibits adipogenic differentiation and induces trans-differentiation into hepatocyte-like cells in 3T3-L1 preadiocytes
Source: BMC Res Notes. 2022 May 10;15:164. doi: 10.1186/s13104-022-06051-x (PMC9092885; doi:10.1186/s13104-022-06051-x)
Supplement: Supplementary file 1 — Additional file 1: Table S1. Primer information. [file 13104_2022_6051_MOESM1_ESM.docx]

**Table S1**. Primer information

| Gene | Primer sequence | | Size  (bp) |
| --- | --- | --- | --- |
|  | Forward | Reverse |  |
| Mest | 5'-AACCGCAGAATCAACCTGCT-3' | 5'-CGAAGAAATTCATGAGCCTGG-3' | 223 |
| ALB | 5'- TGAAGTTGCCAGAAGACATCC-3' | 5'-CAATGCTTTCTCCTTCACACCA-3' | 150 |
| AFP | 5'-TCCTCCTGCTACATTTCGCTG-3' | 5'-CGGAACAAACTGGGTAAAGGTG-3 | 139 |
| 36B4 | 5'-GAGATTCGGGATATGCTGTTGG-3' | 5'-GTTGTCAAACACCTGCTGGATG-3' | 297 |
