# Supplementary material for: Mouse mesoderm-specific transcript inhibits adipogenic differentiation and induces trans-differentiation into hepatocyte-like cells in 3T3-L1 preadiocytes
Source: BMC Res Notes. 2022 May 10;15:164. doi: 10.1186/s13104-022-06051-x (PMC9092885; doi:10.1186/s13104-022-06051-x)
Supplement: Supplementary file 2 — Additional file 2: Table S2. Sequence information of siRNAs targeting mouse Mest. [file 13104_2022_6051_MOESM2_ESM.docx]

**Table S2**. Sequence information of siRNAs targeting mouse Mest

| Gene | siRNA sequence | |
| --- | --- | --- |
|  | sense | antisense |
| siRNA 1 | 5’-UGGUCAUCCAGAAUCGACACUGUGG-3’ | 5’-CCACAGUGUCGAUUCUGGAUGACCA-3’ |
| siRNA 2 | 5’-GAGGAUCCCAUGGGCUUCUUG AAUG-3’ | 5’-CAUUCAAGAAGCCCAUGGGAUCCUC-3’ |
| Negative  control | 5’-UGGGUUCUACAGACGCUACACAUGG-3’ | 5’-CCAUGUGUAGCGUCUGUAGAACCCA-3’ |
