# Supplementary figures and images for: Mouse mesoderm-specific transcript inhibits adipogenic differentiation and induces trans-differentiation into hepatocyte-like cells in 3T3-L1 preadiocytes
Source: BMC Res Notes. 2022 May 10;15:164. doi: 10.1186/s13104-022-06051-x (PMC9092885; doi:10.1186/s13104-022-06051-x)

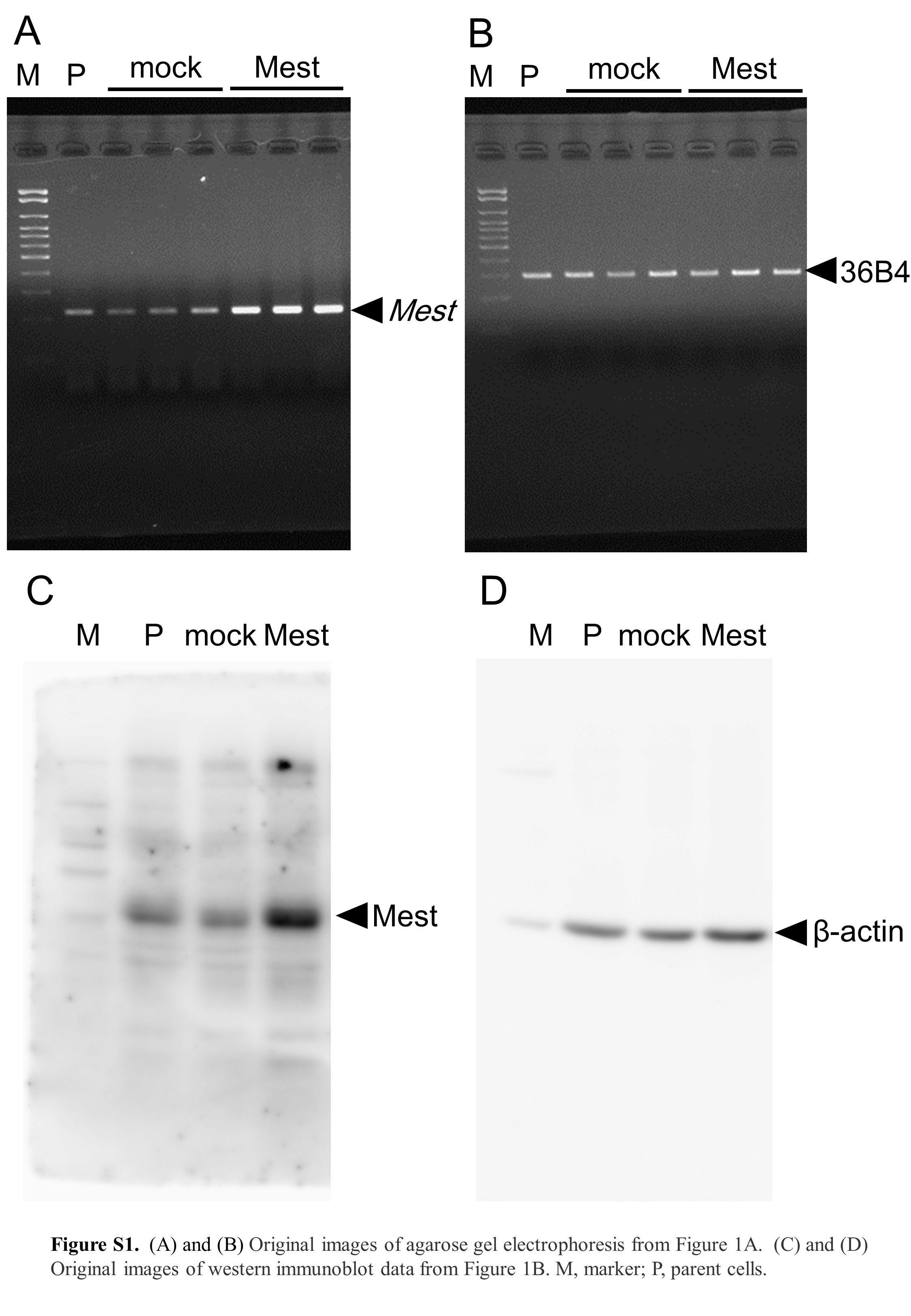

Supplement: Supplementary file 3 — Additional file 3: Figure S1. (A) and (B) Original images of agarose gel electrophoresis from Figure 1 A.(C) and (D) Original images of western immunoblot data from Figure l B. M, marker; P, parent cells. [file 13104_2022_6051_MOESM3_ESM.tif]

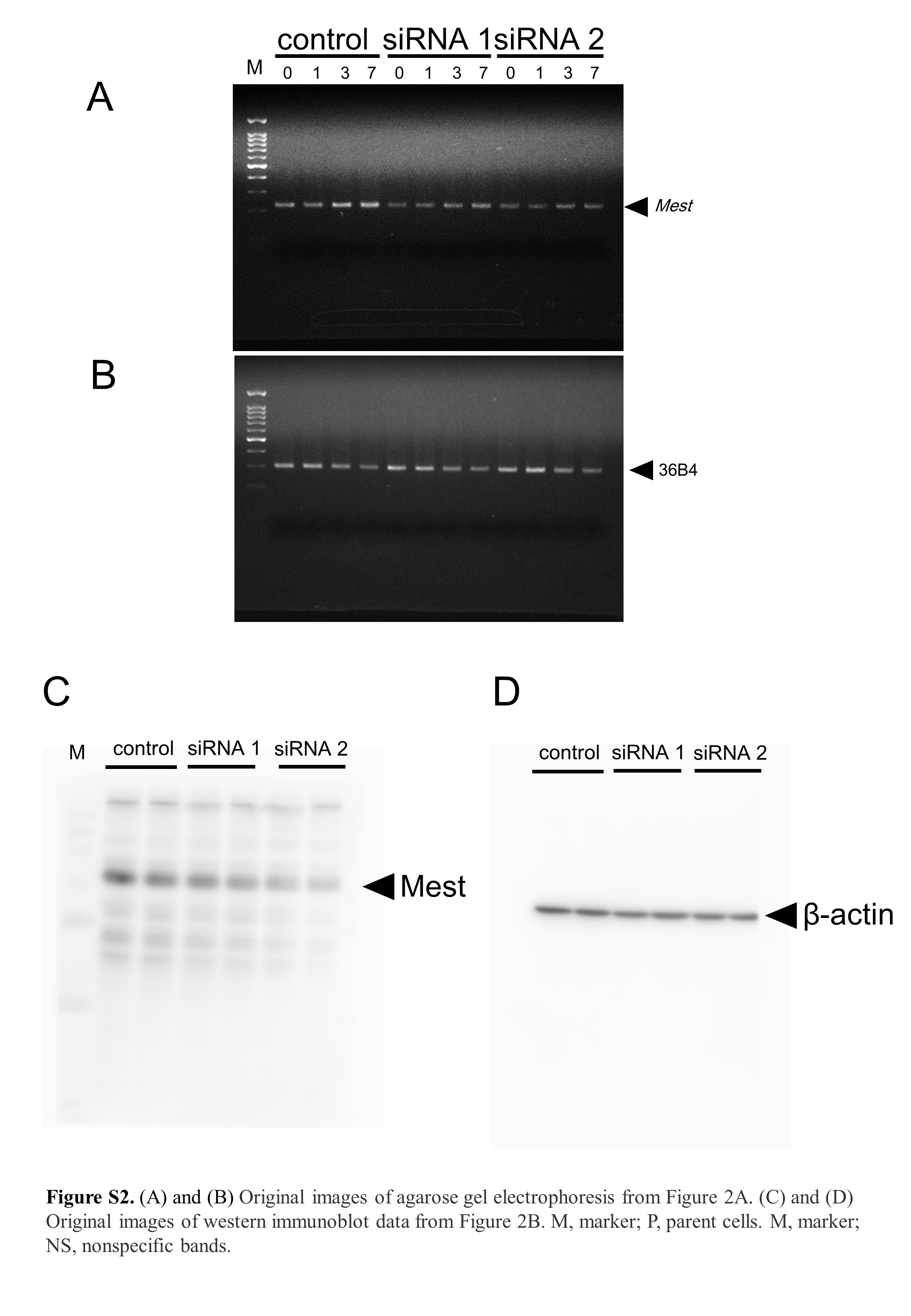

Supplement: Supplementary file 4 — Additional file 4: Figure S2. (A) and (B) Original images of agarose gel electrophoresis from Figure 2A. (C) and (D) Original images of western immunoblot data from Figure 2B. M, marker; P, parent cells. M, marker; NS, nonspecific bands. [file 13104_2022_6051_MOESM4_ESM.tif]

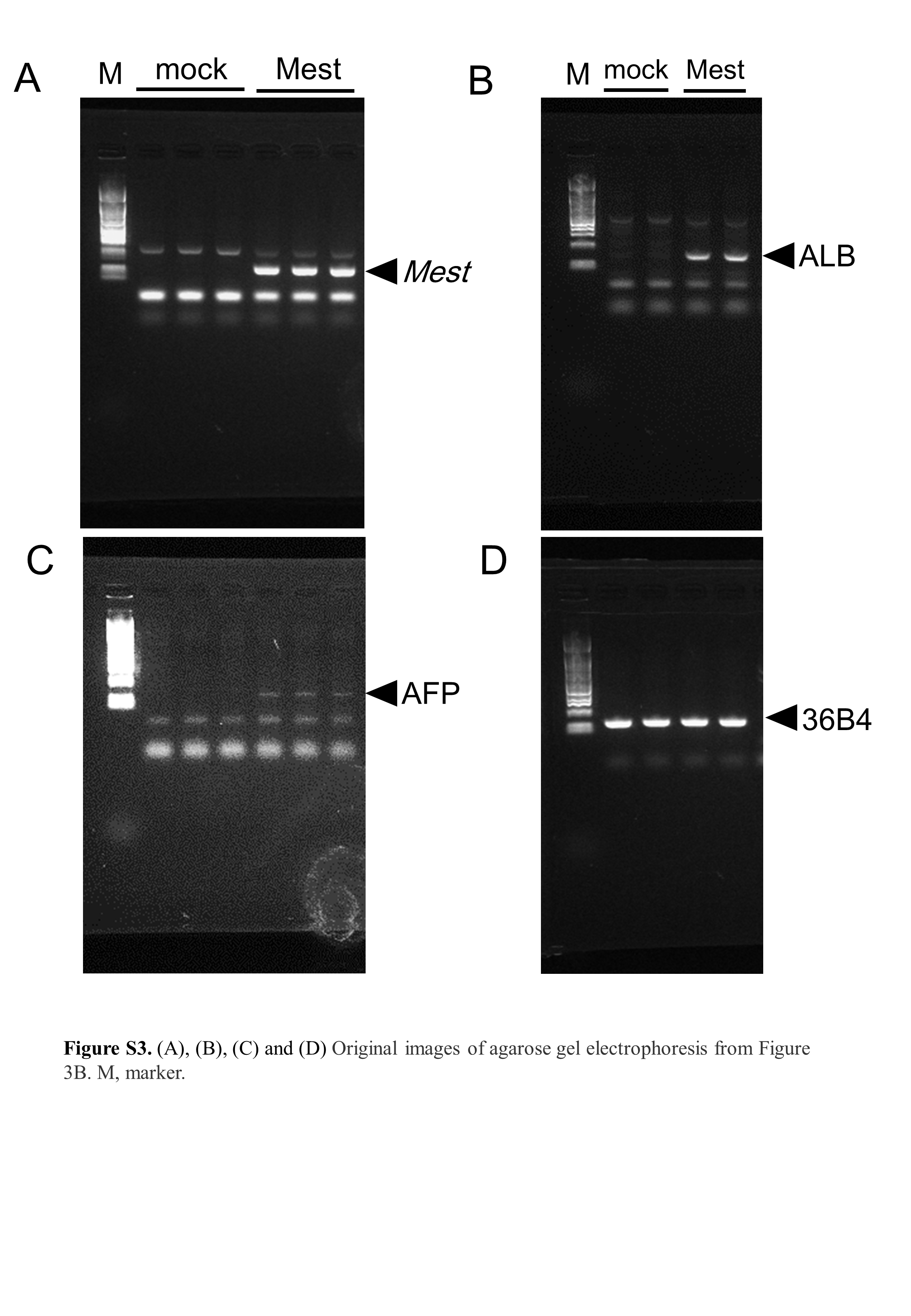

Supplement: Supplementary file 5 — Additional file 5: Figure S3. (A), (B), (C) and (D) Original images of agarose gel electrophoresis from Figure 3B. M, marker. [file 13104_2022_6051_MOESM5_ESM.tif]
